# Grouping of Emergency Department-based Cardiac Arrest Patients According to Clinical Features to Assess Patient Outcomes

**DOI:** 10.5811/westjem.46556

**Published:** 2025-11-26

**Authors:** Joshua Leow, Po-Chun Shih, Jun-Wan Gao, Chih-Hung Wang, Tsung-Chien Lu, Chien-Hua Huang, Chu-Lin Tsai

**Affiliations:** * University of Tennessee Health Science Center, College of Medicine, Department of Emergency Medicine, Memphis, Tennessee; † National Taiwan University Hospital, Department of Emergency Medicine, Taipei, Taiwan; ‡ National Taiwan University, College of Medicine, Department of Emergency Medicine, Taipei, Taiwan

## Abstract

**Introduction:**

While research has begun to understand emergency department-based cardiac arrest (EDCA), consensus on what exactly constitutes EDCA remains unknown. In this study we aimed to explore the grouping of EDCA by using an unsupervised machine-learning algorithm and to investigate how these underlying clusters related to patient outcomes.

**Methods:**

We retrieved electronic health record data from an ED in a tertiary medical center. The EDCAs were identified via the cardiopulmonary resuscitation log. We used k-means cluster analysis to group EDCAs and t-distributed stochastic neighbor embedding (t-SNE) for visualization. Primary outcomes were ED mortality and ED length of stay (LOS). The analyses were repeated using an independent ED data set, the Medical Information Mart for Intensive Care IV Emergency Department (MIMIC-IV-ED) dataset.

**Results:**

From 2019 to 2022, there were 366 EDCA events. Cluster analysis identified three distinct clusters (Cluster 1 or immediate risk, n=54 [15%]; Cluster 2 or early risk, n=274 [75%]; Cluster 3 or late risk, n=38 [10%]). Cluster 1 patients had the shortest median time to EDCA (< 1 hour), followed by Cluster 2 (3 hours) and Cluster 3 (81 hours). Near cardiac arrest at triage was the most common cause of EDCA in Cluster 1, while respiratory illnesses and sepsis were more common in Cluster 3. The causes of EDCA in Cluster 2 were diverse, with predominantly cardiovascular and neurologic emergencies. The t-SNE revealed farther distances from Cluster 1 to the other two clusters, suggesting its most critical nature. Cluster 3 had the highest mortality (58%), followed by Clusters 1 (48%) and 2 (35%) (P = .01). Cluster 1 had the shortest median LOS (median, 4 hours), while Cluster 3 had the longest LOS (81 hours) (P < .001). In the independent data set, Cluster 1 remained, but Clusters 2 and 3 appeared to merge due to a shorter ED LOS overall.

**Conclusion:**

We identified three novel clusters (immediate, early, and late risk) with distinct patterns in clinical presentation, putative causes of ED-based cardiac arrest, and ED outcomes. Understanding these clinical phenotypes may help develop cluster-specific interventions to prevent EDCA or intervene most appropriately. Cluster 1 patients may benefit from resuscitation efforts, and Clusters 2 or 3 patients can benefit from timely interventions for cardiac, respiratory, and neurologic emergencies. In addition, for patients with prolonged ED boarding, periodic monitoring with an early warning system may prevent a cardiac arrest event.

## INTRODUCTION

In-hospital cardiac arrest (IHCA) continues to be a major clinical problem worldwide with substantial morbidity and mortality.^1^, ^2^ Approximately 300,000 IHCA events occur per year in the United States.^3^ While IHCA events occur in a variety of clinical settings, IHCAs in the emergency department (ED cardiac arrest [EDCA]) are less studied and understood.^4^ Emergency department cardiac arrest appears to differ from other IHCA, and it may be associated with higher survival to hospital discharge (22.8%), as opposed to IHCA in the ward setting (10.8%) or intensive care unit (15.8%).^4^–^8^ There is growing interest in studying the epidemiology of EDCA with a particular focus on the development of tools to identify and predict it.^9^–^12^

While research has begun to understand EDCA, consensus on what exactly constitutes it remains unknown. Emergency department cardiac arrest can be thought of as an umbrella term comprising overlapped individual items in a high-dimensional space. The compositional heterogeneity of EDCA can be studied via cluster analysis, also known as clinical phenotyping. This type of exploratory, unsupervised machine-learning algorithm aims to reveal underlying structures or biological clusters by reducing data dimensions. This technique is unbiased in the sense that subject groupings are made without any previously defined hypothesis or a priori assumption. It has shown promise in the research of other diseases such as diabetes and asthma.^13^, ^14^, Moore et al used unsupervised cluster analysis to identify five distinct clinical phenotypes of asthma, which supports clinical heterogeneity in asthma and the need for new approaches in classifying disease severity in asthma.^14^ To the best of our knowledge, however, unsupervised machine-learning has not been applied in EDCA. Understanding the underlying clusters of EDCA and their relationships with patient outcomes would have implications for risk stratification and early interventions in the ED.

To fill these knowledge gaps, we aimed to explore the EDCA subpopulations by cluster analysis and to investigate how these underlying clusters related to patient outcomes. The two-step approach (unbiased patient characterization and outcome association) also increases the validity of analysis by blinding the outcome to the associational variables, thereby avoiding a data-fishing expedition. We also sought to validate our findings on an independent United States ED dataset.

## METHODS

### Study Design, Setting, and Population

We conducted a retrospective cohort study in the National Taiwan University Hospital (NTUH), a tertiary, academic medical center with approximately 2,400 beds and 100,000 ED visits per year. The ED also manages an observation unit (EDOU), which is staffed by emergency physicians. For quality improvement purposes, the department compiles an EDCA log that includes all EDCAs treated with cardiopulmonary resuscitation (CPR), including those in the EDOU. We defined EDCA as patients who arrived in the ED with vital signs and later developed cardiac arrest in the ED. Thus, out-of-hospital cardiac arrests (OHCA) without return of spontaneous circulation (ROSC) on ED arrival were excluded. The EDCA log contained basic data (demographic and administrative data) from all EDCAs. Additional clinical information was collected through periodic electronic health record (EHR) reviews, with a focus on pre-arrest factors (eg, triage data, structured chief complaints, and pre-arrest vital signs).

**Population Health Research Capsule**

What do we already know about this issue?

*While researchers have begun to understand emergency department cardiac arrest (EDCA), consensus on what exactly constitutes it remains unknown.*

What was the research question?

*What are the distinct clinical subgroups of EDCA, and how are these clusters associated with patient outcomes?*

What was the major finding of the study?

*Cluster 3 (late-risk group) had the highest mortality (58%), followed by Clusters 1 (immediate-risk, 48%) and 2 (early-risk, 35%) (P = .01).*

How does this improve population health?

*The identification by machine-learning of 3 EDCA subgroups with distinct patterns in time to cardiac arrest and ED outcomes suggests time-based specific interventions for each subgroup.*

Data were directly extracted via structured items in the five-level Taiwan Triage and Acuity Scale (TTAS) embedded in our EHR system (not abstracted from hard-copy medical records). The computerized TTAS triage software, adapted from the Canadian Triage and Acuity Scale, has been used for ED triage in Taiwan since 2010. In this study we followed the methods of medical record review studies in emergency medicine research except for interrater reliability assessments.^15^ Putative etiologies included six major categories (cardiovascular, respiratory, sepsis, trauma, near-arrest on arrival, and other) that contained 29 presumed causes of cardiac arrest (online Supplementary Table). The causes of arrest were initially assigned by resident reviewers and finalized by a board-certified attending physician. The medical record abstractors and physician reviewers of arrest etiology were blinded to study outcomes. Monthly EDCA meetings were held to review EDCA cases and discuss possible preventive strategies on a systemic level. We used the EDCA database from its inception (January 1, 2019) to December 31, 2022 and focused on adults ≥ 18 years of age.

### Ethical Approval Statement

This study was approved by the NTUH Institutional Review Board (reference number: 202304105RINC), which waived the requirement for patient informed consent.

### Variables

Patient demographics and time-stamped clinical information at triage were collected, including chief complaint on presentation, mode of arrival, vital signs (temperature, heart rate, systolic and diastolic blood pressure, respiratory rate, oxygen saturation), and levels of consciousness as defined by the Glasgow Coma Scale (GCS). At ED triage, when assessing a patient’s level of consciousness, the triage nurse also recorded whether there was an acute change from the patient’s baseline status. Pain scores were evaluated on a numeric rating scale (NRS) of 0–10, with 0 being no pain and 10 being the worst pain imaginable. The TTAS contained information on 179 structured chief complaints. Based on computerized algorithms, the TTAS classified patients in the following order of acuity: level 1, resuscitation; level 2, emergent; level 3, urgent; level 4, less urgent; and level 5, non-urgent. The TTAS has been validated against hospitalization, ED LOS, and resource use. In our ED, for location-of-care purposes, patients were assigned a subdivision for care after triage, including non-trauma, trauma, and major trauma/resuscitation areas. We classified ED shifts as day (07 am −2:59 pm), evening (3 pm −10:59 pm), and night (11 pm – 6:59 am) shifts. The time interval from triage to CPR was also calculated. For patients who required multiple CPR attempts due to re-arrest, their records were counted only once (first CPR in the ED). Of note, the vital signs for patients with near-cardiac arrest at triage were set to zero due to being rushed to resuscitation without triage measurements.

### Independent Data Set to Validate the Clustering Approach

The Medical Information Mart for Intensive Care IV Emergency Department (MIMIC-IV-ED) dataset is a large, freely available database of ED visits at the Beth Israel Deaconess Medical Center (Boston, MA, USA) between 2011–2019. The database contains approximately 425,000 ED stays. Triage information (eg,, chief complaints and vital signs), diagnostic codes, medication administration, and ED disposition were available. For validation purposes, we included adult patients with a diagnostic code of cardiac arrest (primary or secondary codes). Of them, we further excluded patients with a mention of “arrest” in their chief complaint (presumably OHCA) to arrive at the EDCA population of interest. The MIMIC-IV-ED dataset was not as granular as our own ED data, as some information was not available (eg, time to CPR or seasonality) or not derivable (etiology of arrest).

### Outcome Measures

Primary outcomes were mortality in the ED and ED LOS. The secondary outcome was the putative cause of cardiac arrest.

### Statistical Analysis

We used an unsupervised machine-learning algorithm, k-means with Euclidean distance, to group EDCAs. For this k-means cluster analysis, patient characteristics included demographics (age and sex) and pre-arrest information (triage vital signs, model of arrival, Glasgow Coma Score, pain score, subdivision of care, chief complaint, ED presentation [season, weekend, time of day], and time to CPR). We checked the variance inflation factor (VIF) for all features in the clustering algorithm. Multicollinearity did not seem to be a concern since all VIFs were < 10. The k-means clustering algorithm is an iterative procedure that partitions data into k clusters. The goal of this algorithm was to minimize the sum of squared distance in each cluster, thereby achieving high similarity within clusters. The procedure began with a random selection of k patients as the initial cluster center. Afterward, observations were assigned to the cluster with the closest center, and the cluster centroid was subsequently updated. The process was repeated until all observations remained in the same cluster from the previous iteration. The optimal number of clusters was determined by a large reduction in the sum of squared distances (ie, elbow statistic).^16^ During the entire clustering process, the algorithm was blinded to outcome variables. The clustering procedure was first performed using the NTUH dataset and then repeated using the MIMIC-IV-ED dataset for validation purposes.

Summary statistics are presented as proportions (with 95% CI), means (with standard deviations), or medians (with interquartile ranges). To compare differences between clusters, we examined bivariate associations using Student *t*-tests, Mann-Whitney tests, chi-square tests, and chi-square trend tests, as appropriate. To visualize the underlying patterns in the multi-dimensional data, we used the 3-D *t*-distributed stochastic neighbor embedding (*t*-SNE). The *t*-SNE is a commonly used non-linear dimensionality reduction technique.^17^ We plotted time to CPR using a violin plot for visualization purposes. The violin plot shows the full distribution of the data, usually smoothed by a kernel probability density estimator.

All analyses were performed using Stata 16.0 software (StataCorp, College Station, TX) and the SAS Viya software platform (SAS Institute Inc, Cary, NC). All *P*-values are two-sided, with *P* < .05 considered statistically significant.

## RESULTS

From 2019 to 2022, there were 368 EDCAs. For the current analysis, we excluded two children < 18 years of age, leaving a cohort of 366 adult patients. Table 1 shows the clinical characteristics of the study cohort. Overall, the mean age of these patients was 72 years, and 40% were women. The vast majority of the EDCAs were initially assigned to the non-trauma area, and 45% arrived by ambulance. A variety of chief complaints were recorded on ED presentation, with dyspnea being the most common. The triage levels were quite high, with 34% triaged to level 1. The initial mean values of vital signs and consciousness level were deranged, with a wide range of variation presented in Table 2. Most patients were pain-free. The median time from triage to CPR was only three hours. Approximately 13% of the patients’ arrest rhythm was shockable, 8% was asystole, and 79% was pulseless electrical activity (PEA).

As shown in the Online Supplementary Figure, the clustering algorithm identified three clusters as there was a kink in the within sum of squares at k = 3 (number of clusters). Of these 366 patients experiencing IHCA, 54 patients were organized into Cluster 1 (14.8%), 274 patients in Cluster 2 (74.9%), and 38 patients in Cluster 3 (10.4%) (Table 2). The three clusters did not differ with regard to age, sex, presenting season, weekend, time of the day, subdivision, or mode of arrival. In terms of chief complaint upon presentation to the ED, Cluster 1 had more complaints of change in consciousness or injury, Cluster 2 had more complaints of chest pain or fever, and Cluster 3 had more complaints of dyspnea or abdominal pain. Furthermore, Cluster 1 patients were mostly triaged to level 1, Cluster 2 to level 2, and Cluster 3 to level 3.

**Figure 1:**
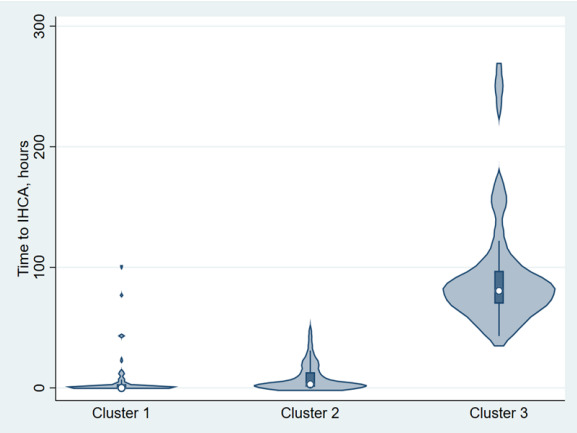
Violin plots showing the distribution of time to in-hospital cardiac arrest requiring cardiopulmonary resuscitation by cluster. Cluster 1 represents an immediate risk, Cluster 2 represents an early risk, and Cluster 3 represents a late risk. For each cluster, the median is shown as a circle, and the first-to-third interquartile range is shown as a shaded box. *IHCA*, in-hospital cardiac arrest.

**Figure 2:**
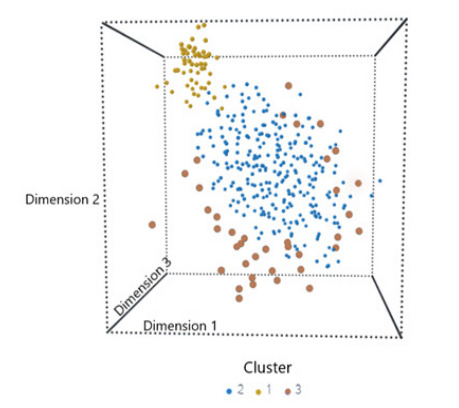
The t-SNE plot. Each circle represents a patient. The t-SNE visualizes the three clusters of data in the three-dimensional space, revealing farther distances from Cluster 1 (upper left corner, immediate risk) to the other two clusters (Cluster 2, early risk; Cluster 3, late risk). The plotted dimensions are abstract and don’t correspond to original features. They are latent dimensions that best preserve local similarities and show distances between observations. *t-SNE*, t-distributed stochastic neighbor-embedding.

Vital signs also reflected the different levels of acuity across the three clusters; for example, Cluster 1 had highly abnormal vital signs. The trend of decreasing acuity from Cluster 1 to 3 appeared the same in relation to the GCSlasgow Coma Scale and acute change in consciousness. Most patients were pain-free, although there was a slight difference across clusters. Cluster 2 had a higher rate of shockable rhythm at arrest, but this was not statistically significant. An important clinical difference across the three clusters was time from triage to CPR, with medians of 0, 3, and 80.5 hours for Cluster 1, 2, and 3, respectively. Figure 1 depicts the distributions of time to CPR across the three clusters via a violin plot. In terms of high-dimensional distance measures reduced to a 3-D plot (Figure 2), *t*-SNE revealed much farther distances from Cluster 1 to the other 2 clusters, suggesting its unique and most critical nature.

Table 3 characterizes each cluster based on the putative causes of cardiac arrest. Cluster 1 patients comprised more trauma patients; it also saw the highest rate of OHCA with ROSC or near-IHCA at triage. Cluster 2 comprised a higher prevalence of diagnoses related to the cardiovascular system and a variety of “other” diagnoses (n = 90). The most common diagnoses in the other category were gastrointestinal bleeding (n = 19), advanced cancer with multiple causes (n = 18), cerebrovascular accident (n = 9), hyperkalemia (n = 7), and head and neck cancer bleeding (n = 5). Cluster 3 held the most diagnoses of sepsis and respiratory illnesses (mainly pneumonia).

Table 4 reveals the outcomes of patients assigned to each cluster. Cluster 3 patients had the highest mortality (58%), followed by Clusters 1 (48%) and 2 (35%) (*P* = .01). The ED LOS was longest in Cluster 3 (median, 81 hours), followed by Clusters 2 (median, 6 hours) and 1 (median, 4 hours) (*P* < .001).

### Independent Dataset

From 2011 to 2019, there were 207 EDCAs in the independent dataset MIMIC-IV-ED. Table 5 shows the clinical characteristics of the cohort. Approximately 72% of the patients arrived by ambulance, and 73% were triaged at level 1. After the clustering procedure, only two clusters were identified. A total of 154 patients were organized into Cluster 1 (74%) and 53 patients in Cluster 2 (26%) (Table 6). Cluster 1 patients’ acuity was much higher than Cluster 2, as more patients arrived by ambulance and were triaged at level 1 in Cluster 1. Regarding chief complaints and outcomes (Table 7), Cluster 1 patients comprised patients with a motor vehicle collision, comatose status, or ST-elevation myocardial infarction, and transferred patients. By contrast, Cluster 2 patients comprised patients with dyspnea, chest pain, altered mental status, or syncope. Emergency department mortality was similar between the two groups, with a slightly shorter ED LOS in Cluster 1 patients.

## DISCUSSION

Our exploration into the heterogeneity of 366 patients who experienced in-hospital cardiac arrest in the ED yielded three distinct clinical phenotypes. As a result of unsupervised machine-learning, time to CPR appeared to be the most important feature when characterizing EDCA, and a trimodal temporal pattern was identified. Near-cardiac arrest at ED triage and OHCA with ROSC were the most common causes of EDCA in Cluster 1 patients, while respiratory illness and sepsis were the most common causes of EDCA in Cluster 3 patients. The causes of EDCA in Cluster 2 patients were quite diverse, including cardiovascular diseases and other causes. Regarding outcomes, Cluster 3 patients had the longest ED. LOS and highest mortality. Cluster 1 patients had the second highest mortality and the shortest time to CPR and ED. LOS. Cluster 1 patients also possessed a critical nature that distinguished themselves in the dimensional plot. In the independent dataset, Cluster 1 remained, but Clusters 2 and 3 appeared to merge due to a shorter ED LOS overall.

Resuscitation care is especially important for patients in Cluster 1. This was the subgroup comprising patients with trauma and near-IHCA at triage or OHCA with ROSC. The time from triage to CPR was < one hour, and the mortality rate was quite high. We considered this group to be immediate and very high risk. As stated in the Methods section and Table 2 footnote, the vital signs for patients with near-cardiac arrest at triage were set to zero due to being rushed to resuscitation without triage measurements. As such, the vital signs in this group were highly abnormal. Because of the very short time to cardiac arrest, this group would especially benefit from earlier and intensive interventions. Realistically, within this ultra-short time frame, it is less likely for any of these patients to have undergone detailed laboratory examinations and workup. Immediate resuscitation with Advanced Cardiovascular Life Support (ACLS) may be key to reversing the deteriorating course of these peri-arrest patients.^18^ Combined with the advantages of the ED where there is 24-hour onsite physician coverage and quick access to Advanced Life Support equipment, timely management of imminent EDCA should be possible.^19^

We considered the patients of Cluster 2 as the early- and medium-to-high-risk group. With a median time from CPR to triage of 3 hours, IHCA was impending, but not as quickly as Cluster 1. As a result, there may be more time available for clinical decision-making over the ED stay. The most common putative causes of cardiac arrest in this group are diverse, as results indicated causes ranging across the gastrointestinal system, stroke, and cancer, among others. Additionally, because the patients in this cluster presented with somewhat normal vital signs, the question could be asked: why do these patients deteriorate somewhat acutely? The subtle clues may be buried in the initial clinical presentation of the patient at triage. For example, are there subtle physical or vital signs suggesting imminent gastrointestinal or head and neck cancer bleeding that could subsequently lead to shock or airway issues? Are there unrecognized neurologic signs indicating a life-threatening stroke?

This cluster could benefit from machine-learning algorithms that incorporate large amounts of traditional and unconventional data (physical examination images or videos) to detect clinical deterioration. For example, our recent research used triage ECG images to accurately detect EDCA seven hours prior to arrest.^20^ Another study used video at triage to predict hospitalization via computer vision algorithms.^21^ Further research and data collection can more clearly define identifying characteristics in a time-sensitive clinical setting.

The patients of Cluster 3 saw deterioration to EDCA within approximately three days of ED arrival; we considered this cluster as the late EDCA group. In Taiwan, these patients are classified as “ED boarders” awaiting inpatient beds, many of whom are nursing home residents. These patients often had multiple comorbidities and required more coordination effort in finding an inpatient bed. Overall, the median ED LOS during the study period for patients who were admitted through the ED was 23 hours, which was much shorter than that for this boarder population (80.5 hours). These patients presented to the ED with largely normal vital signs and were initially assigned to a less severe triage level. Data indicates that the most common putative causes of cardiac arrest for patients in this cluster were sepsis and pneumonia, consistent with the common infectious causes of IHCA on the ward.^22^ Furthermore, with a greater time frame from triage to CPR, this cluster may benefit greatly from periodic monitoring (eg, early warning system) to prevent a cardiac arrest event. The major difference between inpatient IHCA and the “boarder EDCA” populations was less frequent monitoring and more staff handovers, likely leading to the highest mortality in the cluster. We previously developed a deep learning-based early warning score that may be helpful in detecting EDCA in advance.^23^ Nonetheless, this boarder EDCA population was less relevant to other EDs with a shorter LOS, as indicated by this cluster being no longer relevant in the US MIMIC-ED data set.

With a preliminary understanding of the three novel EDCA clusters, the prospect of developing a more concrete consensus on what constitutes EDCA could become a reality. Current guidelines on IHCA may benefit from a separate section on EDCA, considering its diverse patient population of both time-sensitive critical conditions and a variety of initially stable medical conditions that may later deteriorate in the ED.^24^, ^25^ Specifically, Cluster 2 patients deserve more research as there was still a three-hour time window to prevent deterioration in this patient population with diverse causes of EDCA. In the trauma literature, a trimodal distribution of trauma deaths has been described based on the time interval from injury to death.^26^

Similar to this concept, we proposed a trimodal EDCA framework (immediate, early, and late) for the three clusters of EDCA based on the time interval from ED arrival to CPR. For EDs with fewer boarding issues, this trimodal framework may reduce to a bimodal (immediate and early) pattern. We feel that the goal for the independent dataset is to test the clustering approach in a different patient population, and a slightly different pattern, or decreased model performance, should be acceptable.

## LIMITATIONS

This study has some potential limitations. First, this research was limited by including two medical centers in different countries. To replicate the three phenotypes identified in our study, further research needs to be conducted in a variety of different EDs with more recent data. Second, to date there has been no consensus on the definition of EDCA.^27^ We arbitrarily included OHCA cases with ROSC because they arrived in the ED with vital signs and were likely to re-arrest in the ED. In addition, this population was likely excluded from the independent dataset. Perhaps future expert meetings need to consider the formal definition of EDCA for research standardization purposes. Finally, unsupervised machine-learning has some disadvantages, including the lack of a priori knowledge and no objective evaluation metrics. However, we have carefully pre-selected relevant features and attempted to interpret the findings using existing knowledge.

## CONCLUSION

By way of unsupervised machine learning algorithms via cluster analysis, we identified and characterized three distinct ED cardiac-arrest phenotypes through the perspective of different pre-arrest variables. Quantifying similarities and dissimilarities of high-dimensional data allowed us to identify the three clusters, thereby performing subsequent outcome association. Simply put, time is of the essence when characterizing EDCA, and a trimodal temporal pattern was identified. Namely, an ED patient may be at immediate, early, and late risk for EDCA during his/her ED stay. A better understanding of these clinical phenotypes may help develop cluster-specific and time-appropriate intervention strategies to avoid EDCA and patient deaths. Specifically, Cluster 1 patients may benefit from resuscitation coordinated by an excellent resuscitation team. To prevent clinical deterioration in Cluster 2 or 3 patients, emergency clinicians should familiarize themselves with early clues in cardiac, pulmonary, and neurologic emergencies to provide timely interventions for these EDCA-prone conditions. In addition, for patients with prolonged ED boarding, periodic monitoring with an early warning system may prevent a cardiac arrest event.

## Supplementary Information





## Figures and Tables

**Table 1:** Baseline clinical characteristics of emergency department patietns with cardiac arrest.

Variable	N = 366
Age, mean (SD), yr	72.0 (15.5)
Female sex, n (%)	146 (39.9)
Season, n (%)	
Spring (Mar. – May)	93 (25.4)
Summer (Jun. – Aug.)	90 (24.6)
Fall (Sep. – Nov.)	98 (26.8)
Winter (Dec. – Feb.)	85 (23.2)
Weekend, n (%)	104 (28.4)
Presenting Time, n (%)	
7 AM – 2:59 PM	153 (41.8)
3 PM – 10:59 PM	138 (37.7)
11 PM – 6:59 AM	75 (20.5)
Subdivision, n (%)	
Non-trauma	344 (94.0)
Trauma	10 (2.7)
Major trauma	12 (3.3)
Arrival by ambulance, n (%)	166 (45.4)
Most common chief complaint, n (%)	
Dyspnea	90 (24.6)
Chest pain	49 (13.4)
Fever	34 (9.3)
Consciousness change	24 (6.6)
Injury	20 (5.5)
Abdominal pain	18 (4.9)
Triage level, n (%)	
1 (highest acuity)	123 (33.6)
2	131 (35.8)
3	110 (30.1)
4	2 (0.6)
5 (lowest acuity)	0 (0)
Vital sign at triage	
Systolic blood pressure, mean (SD), mm Hg	105.5 (53.5)
Diastolic blood pressure, mean (SD), mm Hg	61.9 (31.0)
Heart rate, mean (SD), beats per minute	88.3 (35.4)
Body temperature, mean (SD), °C	36.4 (3.6)
Respiratory rate, mean (SD), breaths per minute	20.5 (7.3)
Oxygen saturation, median (IQR), %	97 (94–99)
Glasgow Coma Scale, mean (SD)	
Eye	3.4 (1.1)
Verbal	3.8 (1.8)
Motor	5.0 (1.8)
Acute change of consciousness, n (%)	170 (46.5)
Pain score (0–10),median (IQR), %	0 (0–0)
Time from triage to CPR, median (IQR), hour	3 (0–19)
Shockable arrest rhythm, n (%)	48 (13.1)

*CPR*, cardiopulmonary resuscitation; *IQR*, interquartile range; *mm Hg*, millimeters of mercury.

**Table 2:** Patient characteristics of each cluster signifying risk of cardiac arrest.

Variable	Cluster 1 (n = 54)	Cluster 2 (n = 274)	Cluster 3 (n = 38)	P-value
Age, mean (SD), yr	70.7 (16.1)	71.9 (15.5)	77.7 (14.0)	.07
Female sex, n (%)	19 (35.2)	111 (40.5)	17 (44.7)	.64
Season, n (%)				.79
Spring (Mar. – May)	13 (24.1)	71 (25.9)	9 (23.7)	
Summer (Jun. – Aug.)	9 (16.7)	72 (26.3)	9 (23.7)	
Fall (Sep. – Nov.)	17 (31.5)	71 (25.9)	10 (26.3)	
Winter (Dec. – Feb.)	15 (27.8)	60 (21.9)	10 (26.3)	
Weekend, n (%)	20 (37.0)	72 (26.3)	12 (31.6)	.25
Presenting Time, n (%)				.08
7 AM – 2:59 PM	26 (48.2)	106 (38.7)	21 (55.3)	
3 PM – 10:59 PM	17 (31.5)	106 (38.7)	15 (39.5)	
11 PM – 6:59 AM	11 (20.4)	62 (22.6)	2 (5.3)	
Subdivision, n (%)				.44
Non-trauma	50 (92.6)	256 (93.4)	38 (100.0)	
Trauma	1 (1.9)	9 (3.3)	0 (0)	
Major trauma	3 (5.6)	9 (3.3)	0 (0)	
Arrival by ambulance, n (%)	32 (59.3)	120 (43.8)	14 (36.8)	.06
Most common chief complaint, n (%)				.02
Dyspnea	13 (24.1)	65 (23.7)	**12 (31.6)**	
Chest pain	7 (13.0)	**39 (14.2)**	3 (7.9)	
Fever	1 (1.9)	**29 (10.6)**	4 (10.5)	
Consciousness change	**9 (16.7)**	13 (4.7)	2 (5.3)	
Injury	**6 (11.1)**	14 (5.1)	0 (0)	
Abdominal pain	0 (0)	15 (5.5)	**3 (7.9)**	
Other	18 (33.3)	99 (36.1)	**14 (36.8)**	
Triage level, n (%)				< .001
1 (highest acuity)	**37 (68.5)**	81 (29.6)	5 (13.2)	
2	7 (13.0)	**114 (41.6)**	10 (26.3)	
3	10 (18.5)	77 (28.1)	**23 (60.5)**	
4	0 (0)	**2 (0.7)**	0 (0)	
5 (lowest acuity)	0 (0)	0 (0)	0 (0)	
Vital sign at triage*				
Systolic blood pressure, mean (SD), mm Hg	3.7 (13.5)	**121.4 (34.9)**	136.1 (31.8)	< .001
Diastolic blood pressure, mean (SD), mm Hg	2.4 (8.7)	**71.2 (19.9)**	79.7 (18.5)	< .001
Heart rate, mean (SD), beats per minute	**44.4 (51.2)**	96.3 (25.8)	92.8 (19.9)	< .001
Body temperature, mean (SD), °C	**34.2 (8.5)**	36.8 (1.5)	36.9 (0.8)	.003
Respiratory rate, mean (SD), breaths per minute	**11.2 (11.4)**	22.1 (4.9)	22.1 (4.1)	< .001
Oxygen saturation, median (IQR), %	0 (0–95)	**97 (95–99)**	97 (96–98)	< .001
Glasgow Coma Scale, mean (SD)				
Eye	**1.8 (1.2)**	3.7 (0.8)	3.9 (0.5)	< .001
Verbal	1.5 (1.5)	4.2 (1.5)	**4.3 (1.6)**	< .001
Motor	**2.3 (2.0)**	5.5 (1.3)	5.6 (0.9)	< .001
Acute change of consciousness, n (%)	**45 (83.3)**	110 (40.2)	15 (39.5)	< .001
Pain score (0–10), median (IQR), [range]	0 (0–0), [0–8]	**0 (0–0), [0–10]**	0 (0–0), [0–8]	.03
Shockable arrest rhythm, n (%)	7 (13.0)	40 (14.6)	1 (2.6)	.12
Time from triage to CPR, median (IQR), hour	0 (0–3)	3 (1–13)	**80.5 (70.0–97.0)**	< .001

Significant differences with the highest percentage/value are highlighted in bold.

* The vital signs for patients with near cardiac arrest at triage were set to zero due to being rushed to resuscitation without triage measurements.

*CPR*, cardiopulmonary resuscitation; *IQR*, interquartile range; *mm Hg*, millimeters of mercury.

**Table 3:** Putative cause of cardiac arrest.

	Cluster 1 (immediate risk) (n=54)	Cluster 2 (early risk)(n=274)	Cluster 3 (late risk)(n=38)	P-value
Diagnosis				<0.001
CV	13 (24.1)	**73 (26.6)**	4 (10.5)	
Sepsis	6 (11.1)	2 (11.7)	**10 (26.3)**	
Respiratory	11 (20.4)	59 (21.5)	**15 (39.5)**	
Trauma	**4 (7.4)**	14 (5.1)	0 (0.0)	
Other	12 (22.2)	**90 (32.9)**	9 (23.7)	
OHCA with ROSC or near IHCA at triage	**8 (14.8)**	6 (2.2)	0 (0.0)	

Significant differences with the highest percentage/value were highlighted in bold.

*CV*, cardiovascular; *IHCA*, in-hospital cardiac arrest; *OHCA*, out-of-hospital cardiac arrest; *ROSC*, return of spontaneous circulation.

**Table 4:** Study outcomes by cluster.

	Cluster			
Outcome	1 (immediate risk) (n = 54)	2 (early risk) (n = 274)	3 (late risk) (n = 38)	*P*-value
Mortality, n (%)	26 (48.2)	97 (35.4)	**22 (57.9)**	0.01
ED length of stay, median (IQR), hour	4.0 (3.0–7.0)	6.0 (3.0–20.0)	**80.5 (70.0–93.0)**	<0.001

Significant differences with the highest percentage/value are highlighted in bold.

*ED*, emergency department; *IQR*, interquartile range.

**Table 5:** Baseline clinical characteristics of emergency department patients with cardiac arrest in the Medical Information Mart for Intensive Care-IV Emergency Department dataset.

Variable	N = 207
Age, mean (SD), yr	64.2 (20.9)
Female sex, n (%)	91 (44.0)
Arrival by ambulance, n (%)	149 (72.0)
Most common chief complaint, n (%)	
Abdominal pain	4 (1.9)
Fever	2 (1.0)
Dyspnea	12 (5.8)
Dizziness	1 (0.5)
Chest pain	12 (5.8)
Other	176 (85.0)
Emergency Severity Index, n (%)	
1 (highest acuity)	152 (73.4)
2	47 (22.7)
3	8 (3.9)
4	0 (0)
5 (lowest acuity)	0 (0)
Vital sign at triage*	
Systolic blood pressure, mean (SD), mm Hg	32.2 (55.3)
Diastolic blood pressure, mean (SD), mm Hg	18.4 (32.4)
Heart rate, mean (SD), beats per min	21.1 (38.2)
Body temperature, mean (SD), °C	8.0 (15.1)
Respiratory rate, mean (SD), breaths per min	4.9 (8.9)
Oxygen saturation, median (IQR), %	0 (0–0)
Glasgow Coma Scale < 15, n (%)	37 (17.9)
Pain score (0–10), median (IQR), %	0 (0–0)

* The vital signs for patients with near cardiac arrest at triage were set to zero due to being rushed to resuscitation without triage measurements

*SD*, standard deviation; *IQR*, interquartile range, *mm Hg*, millimeters of mercury.

**Table 6:** Patient characteristics of each cluster in the Medical Information Mart for Intensive Care-IV Emergency Department dataset.

Variable	Cluster 1 (n = 154)	Cluster 2 (n = 53)	P-value
Age, mean (SD), yr	**61.7 (21.4)**	71.6 (17.8)	< .001
Female sex, n (%)	65 (42.2)	26 (49.1)	.39
Arrival by ambulance, n (%)	**119 (77.3)**	30 (56.6)	.004
Most common chief complaint, n (%)			< .001
Abdominal pain	0 (0.0)	**4 (7.6)**	
Fever	1 (0.7)	**1 (1.9)**	
Dyspnea	5 (3.3)	**7 (13.2)**	
Dizziness	0 (0.0)	**1 (1.9)**	
Chest pain	7 (4.6)	**5 (9.4)**	
Other	**141 (91.6)**	35 (66.0)	
Emergency Severity Index, n (%)			< .001
1 (highest acuity)	**143 (92.9)**	9 (17.0)	
2	11 (7.1)	**36 (67.9)**	
3	0 (0.0)	**8 (15.1)**	
4	0 (0.0)	0 (0.0)	
5 (lowest acuity)	0 (0.0)	0 (0.0)	
Vital sign at triage*			
Systolic blood pressure, mean (SD), mm Hg	1.3 (9.3)	**122.2 (27.7)**	< .001
Systolic blood pressure, mean (SD), mm Hg	0.5 (4.5)	**70.5 (19.6)**	< .001
Heart rate, mean (SD), beats per minute	0.9 (7.8)	**80.0 (28.9)**	< .001
Body temperature, mean (SD), °C	0 (0.0)	**31.1 (13.2)**	< .001
Respiratory rate, mean (SD), breaths per minute	0.4 (3.1)	**18.1 (6.9)**	< .001
Oxygen saturation, median (IQR), %	0 (0–0)	**97 (94–99)**	< .001
Glasgow Coma Scale < 15, n (%)	31 (20.1)	6 (11.3)	.15
Pain score (0–10), median (IQR), [range]	0 (0–0)	**0 (0–2)**	< .001

Significant differences with the highest percentage/value were highlighted in bold.

* The vital signs for patients with near cardiac arrest at triage were set to zero due to being rushed to resuscitation without triage measurements.

*IQR*, interquartile range; *mm Hg*, millimeters of mercury.

**Table 7:** Chief complaints and study outcomes by cluster in the Medical Information Mart for Intensive Care-IV Emergency Department dataset.

	Cluster 1 (n = 154)		Cluster 2 (n = 53)	
Chief complaint free text (most common)				
MVC	35 (22.7)	Dyspnea	9 (17.0)	
Transfer	28 (18.2)	Chest pain	7 (13.2)	
Coma	21 (13.6)	Altered mental status	7 (13.2)	
STEMI	18 (11.7)	Syncope	7 (13.2)	
Outcome				P-value
Mortality, n (%)	56 (36.4)		26 (49.1)	.10
ED length of stay, median (IQR), hour	4.0 (2.5–5.7)		**5.2 (4.0–8.0)**	< .001

Significant differences with the highest percentage/value are highlighted in bold.

*ED*, emergency department; *IQR*, interquartile range, *MVC*, motor vehicle collision; *STEMI*, ST-elevation myocardial infarction.

## References

[1] Andersson A, Arctaedius I, Cronberg T (2022). In-hospital versus out-of-hospital cardiac arrest: characteristics and outcomes in patients admitted to intensive care after return of spontaneous circulation. Resuscitation.

[2] Andersen LW, Holmberg MJ, Berg KM (2019). In-hospital cardiac arrest. JAMA.

[3] Penketh J, Nolan JP (2022). In-hospital cardiac arrest: the state of the art. Crit Care.

[4] Kayser RG, Ornato JP, Peberdy MA (2008). Cardiac arrest in the emergency department: a report from the National Registry of Cardiopulmonary Resuscitation. Resuscitation.

[5] Soar J (2023). In-hospital cardiac arrest. Curr Opin Crit Care.

[6] DiLibero J, Misto K (2021). Outcomes of in-hospital cardiac arrest: a review of the evidence. Crit Care Nurs Clin North Am.

[7] Myat A, Song KJ, Rea T (2018). Out-of-hospital cardiac arrest: current concepts. Lancet.

[8] Gerecht RB, Nable JV (2023). Out-of-hospital cardiac arrest. Emerg Med Clin North Am.

[9] Mitchell OJL, Edelson DP, Abella BS (2020). Predicting cardiac arrest in the emergency department. J Am Coll Emerg Physicians Open.

[10] Tsai CL, Lu TC, Fang CC (2022). Development and validation of a novel triage tool for predicting cardiac arrest in the emergency department. West J Emerg Med.

[11] Lu TC, Wang CH, Chou FY (2023). Machine learning to predict in-hospital cardiac arrest from patients presenting to the emergency department. Intern Emerg Med.

[12] Mir T, Qureshi WT, Uddin M (2022). Predictors and outcomes of cardiac arrest in the Emergency Department and in-patient settings in the United States (2016–2018). Resuscitation.

[13] Ahlqvist E, Storm P, Karajamaki A (2018). Novel subgroups of adult-onset diabetes and their association with outcomes: a data-driven cluster analysis of six variables. Lancet Diabetes Endocrinol.

[14] Moore WC, Meyers DA, Wenzel SE (2010). Identification of asthma phenotypes using cluster analysis in the Severe Asthma Research Program. Am J Respir Crit Care Med.

[15] Worster A, Bledsoe RD, Cleve P (2005). Reassessing the methods of medical record review studies in emergency medicine research. Ann Emerg Med.

[16] Makles A (2012). Stata Tip 110: How to get the optimal k-means cluster solution. Stata J.

[17] Van der Maaten L (2008). Visualizing data using *t*-SNE. J Mach Learn Res.

[18] Merchant RM, Topjian AA, Panchal AR (2020). Part 1: Executive Summary: 2020 American Heart Association Guidelines for Cardiopulmonary Resuscitation and Emergency Cardiovascular Care. Circulation.

[19] Nallamothu BK, Guetterman TC, Harrod M (2018). How do resuscitation teams at top-performing hospitals for in-hospital cardiac arrest succeed? A qualitative study. Circulation.

[20] Lu SC, Chen GY, Liu AS (2025). Deep Learning-Based Electrocardiogram Model (EIANet) to predict emergency department cardiac arrest: development and external validation study. J Med Internet Res.

[21] Ip W, Xenochristou M, Sui E (2024). Hospitalization prediction from the emergency department using computer vision AI with short patient video clips. NPJ Digit Med.

[22] Allencherril J, Lee PYK, Khan K (2022). Etiologies of in-hospital cardiac arrest: a systematic review and meta-analysis. Resuscitation.

[23] Deng YX, Wang JY, Ko CH (2024). Deep learning-based Emergency Department In-hospital Cardiac Arrest Score (Deep EDICAS) for early prediction of cardiac arrest and cardiopulmonary resuscitation in the emergency department. BioData Min.

[24] Neumar RW, Shuster M, Callaway CW (2015). Part 1: Executive Summary. Circulation.

[25] Monsieurs KG, Nolan JP, Bossaert LL (2015). European Resuscitation Council Guidelines for Resuscitation 2015. Resuscitation.

[26] Gunst M, Ghaemmaghami V, Gruszecki A (2010). Changing epidemiology of trauma deaths leads to a bimodal distribution. Proc (Bayl Univ Med Cent).

[27] Moskowitz A, Holmberg MJ, Donnino MW (2018). In-hospital cardiac arrest: are we overlooking a key distinction?. Curr Opin Crit Care.

