# Supplementary material for: Grouping of Emergency Department-based Cardiac Arrest Patients According to Clinical Features to Assess Patient Outcomes
Source: West J Emerg Med. 2025 Nov 26;26(6):1656–66. doi: 10.5811/westjem.46556 (PMC12698154; doi:10.5811/westjem.46556)
Supplement: Supplementary file 1 [file wjem-26-1656-s001.docx]

**Online Supplementary Table 1**. Classification of putative causes of death.

| **Main category (n=6)** | **Subcategory (n=29)** |
| --- | --- |
| CV | Abdominal aortic aneurysm |
|  | Aortic dissection |
|  | Congestive heart failure |
|  | Myocardial infarction |
|  | Ventricular tachycardia/fibrillation |
| Respiratory | Airway obstruction including sputum impaction |
|  | Chronic obstructive pulmonary disease |
|  | Coronavirus disease (COVID) |
|  | Pneumonia |
| Sepsis | Sepsis |
| Near-arrest on arrival | OHCA with ROSC or near IHCA at triage |
| Trauma | Trauma, multiple |
|  | Trauma, brain |
|  | Trauma, chest |
| Other (in descending order of prevalence) | Cancer, multiple potential causes |
|  | Gastrointestinal bleeding |
|  | Hyperkalemia |
|  | Cerebrovascular accident (stroke), ischemic or hemorrhagic |
|  | Head and neck cancer bleeding |
|  | Diabetic ketoacidosis |
|  | Gastrointestinal tract perforation |
|  | Status epilepticus |
|  | Acidosis, not otherwise specified |
|  | Ischemic bowel disease |
|  | Fluid overload, not otherwise specified |
|  | Hepatocellular carcinoma rupture |
|  | Ileus |
|  | Kidney bleeding, non-traumatic |
|  | Unknown |

Abbreviations: CV = cardiovascular diseases; OHCA = out-of-hospital cardiac arrest; ROSC = return of spontaneous circulation; IHCA = in-hospital cardiac arrest.

**Online Supplementary Table 2**. Baseline clinical characteristics of emergency department patients with cardiac arrest in the MIMIC-ED data set.

| **Variable** | **N=207** |
| --- | --- |
| Age, mean (SD), yr | 64.2 (20.9) |
| Female sex, n (%) | 91 (44.0) |
| Arrival by ambulance, n (%) | 149 (72.0) |
| Most common chief complaint, n (%) |  |
| Abdominal pain | 4 (1.9) |
| Fever | 2 (1.0) |
| Dyspnea | 12 (5.8) |
| Dizziness | 1 (0.5) |
| Chest pain | 12 (5.8) |
| Other | 176 (85.0) |
| Triage level, n (%) |  |
| 1 | 152 (73.4) |
| 2 | 47 (22.7) |
| 3 | 8 (3.9) |
| 4 | 0 (0) |
| 5 | 0 (0) |
| Vital sign at triage* |  |
| Systolic blood pressure, mean (SD), mmHg | 32.2 (55.3) |
| Diastolic blood pressure, mean (SD), mmHg | 18.4 (32.4) |
| Heart rate, mean (SD), beats per min | 21.1 (38.2) |
| Body temperature, mean (SD), °C | 8.0 (15.1) |
| Respiratory rate, mean (SD), breaths per min | 4.9 (8.9) |
| Oxygen saturation, median (IQR), % | 0 (0-0) |
| Glasgow coma scale < 15, n (%) | 37 (17.9) |
| Pain score (0-10), median (IQR), % | 0 (0-0) |

*The vital signs for patients with near cardiac arrest at triage were set to zero due to being rushed to resuscitation without triage measurements.

Abbreviations: SD = standard deviation; IQR = interquartile range.

**Online Supplementary Table 3**. Patient characteristics of each cluster in the MIMIC-ED data set.

| **Variable** | **Cluster 1 (n=154)** | **Cluster 2  (n=53)** | **P value** |
| --- | --- | --- | --- |
| Age, mean (SD), yr | 61.7 (21.4) | 71.6 (17.8) | **0.001** |
| Female sex, n (%) | 65 (42.2) | 26 (49.1) | 0.386 |
| Arrival by ambulance, n (%) | 119 (77.3) | 30 (56.6) | **0.004** |
| Most common chief complaint, n (%) |  |  | **<0.001** |
| Abdominal pain | 0 (0.0) | 4 (7.6) |  |
| Fever | 1 (0.7) | 1 (1.9) |  |
| Dyspnea | 5 (3.3) | 7 (13.2) |  |
| Dizziness | 0 (0.0) | 1 (1.9) |  |
| Chest pain | 7 (4.6) | 5 (9.4) |  |
| Other | 141 (91.6) | 35 (66.0) |  |
| Triage level, n (%) |  |  | **<0.001** |
| 1 | 143 (92.9) | 9 (17.0) |  |
| 2 | 11 (7.1) | 36 (67.9) |  |
| 3 | 0 (0.0) | 8 (15.1) |  |
| 4 | 0 (0.0) | 0 (0.0) |  |
| 5 | 0 (0.0) | 0 (0.0) |  |
| Vital sign at triage* |  |  |  |
| Systolic blood pressure,  mean (SD), mmHg | 1.3 (9.3) | 122.2 (27.7) | **<0.001** |
| Diastolic blood pressure,  mean (SD), mmHg | 0.5 (4.5) | 70.5 (19.6) | **<0.001** |
| Heart rate, mean (SD),  beats per min | 0.9 (7.8) | 80.0 (28.9) | **<0.001** |
| Body temperature, mean  (SD), °C | 0 (0.0) | 31.1 (13.2) | **<0.001** |
| Respiratory rate, mean  (SD), breaths per min | 0.4 (3.1) | 18.1 (6.9) | **<0.001** |
| Oxygen saturation,  median (IQR), % | 0 (0-0) | 97 (94-99) | **<0.001** |
| Glasgow coma scale < 15, n (%) | 31 (20.1) | 6 (11.3) | 0.149 |
| Pain score (0-10), median (IQR), [range] | 0 (0-0) | 0 (0-2) | **<0.001** |

*The vital signs for patients with near cardiac arrest at triage were set to zero due to being rushed to resuscitation without triage measurements.

Statistically significant results are highlighted in bold.

Abbreviations: SD = standard deviation; IQR = interquartile range.
