# Supplementary figures and images for: Grouping of Emergency Department-based Cardiac Arrest Patients According to Clinical Features to Assess Patient Outcomes
Source: West J Emerg Med. 2025 Nov 26;26(6):1656–66. doi: 10.5811/westjem.46556 (PMC12698154; doi:10.5811/westjem.46556)

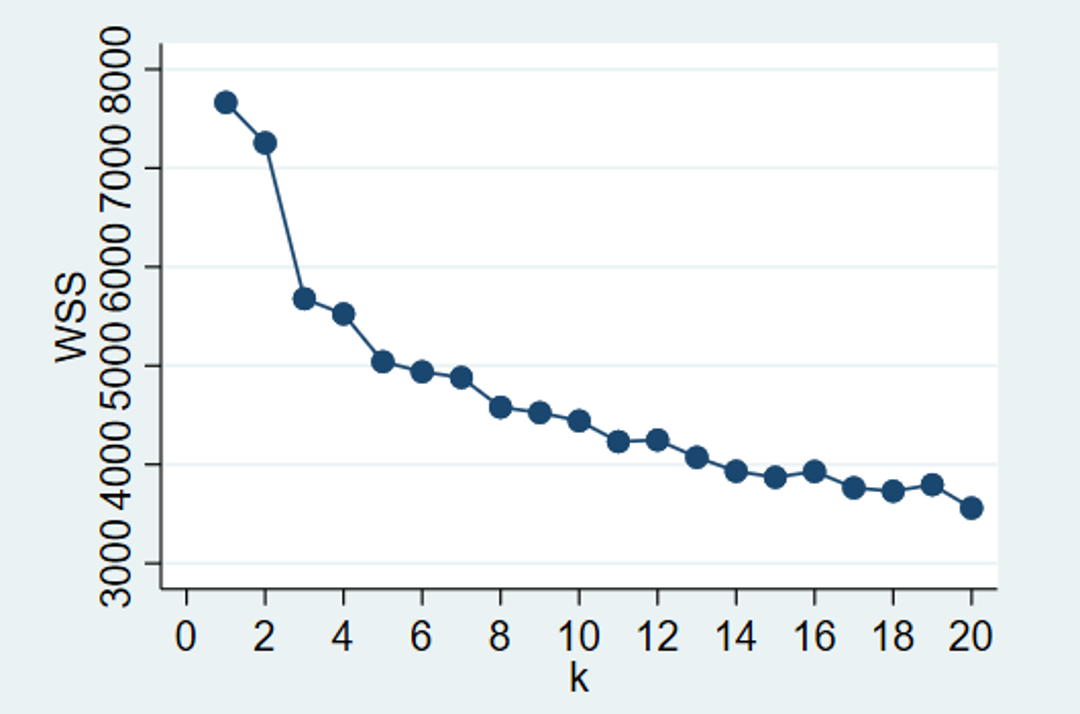

Supplement: Supplementary file 2 [file wjem-26-1656-s002.png]
